# Impacts of Development, Dentofacial Disharmony, and Its Surgical Correction on Speech: A Narrative Review for Dental Professionals

**DOI:** 10.3390/app13095496

**Published:** 2023-04-28

**Authors:** Christine Bode, Nare Ghaltakhchyan, Erika Rezende Silva, Timothy Turvey, George Blakey, Raymond White, Jeff Mielke, David Zajac, Laura Jacox

**Affiliations:** 1 Orthodontics and Oral Surgery Group, Division of Craniofacial and Surgical Care, Adams School of Dentistry, University of North Carolina, 201 Brauer Hall, CB#7450, Chapel Hill, NC 27599-7450, USA; 2 Division of Oral and Craniofacial Health Sciences, Adams School of Dentistry, University of North Carolina, 270 Brauer Hall, CB#270, Chapel Hill, NC 27599-7450, USA; 3 Biological and Biomedical Sciences Program, School of Medicine, University of North Carolina, 1116 Bioinformatics Building, Chapel Hill, NC 25799-7450, USA; 4 Oral and Craniofacial Biomedicine Program, Adams School of Dentistry, University of North Carolina, 365 S Columbia St., Chapel Hill, NC 25799-7450, USA; 5 English Department, North Carolina State University, Tompkins Hall, Raleigh, NC 27695, USA; 6 Speech Pathology Group, Division of Craniofacial and Surgical Care, Adams School of Dentistry, University of North Carolina, 201 Brauer Hall, CB#7450, Chapel Hill, NC 27599-7450, USA

**Keywords:** orthognathic surgery, speech, speech pathology, speech development, craniofacial development, dentofacial disharmony, malocclusion, speech-sound disorders, orthodontics

## Abstract

Speech is a communication method found only in humans that relies on precisely articulated sounds to encode and express thoughts. Anatomical differences in the maxilla, mandible, tooth position, and vocal tract affect tongue placement and broadly influence the patterns of airflow and resonance during speech production. Alterations in these structures can create perceptual distortions in speech known as speech sound disorders (SSDs). As craniofacial development occurs, the vocal tract, jaws, and teeth change in parallel with stages of speech development, from babbling to adult phonation. Alterations from a normal Class 1 dental and skeletal relationship can impact speech. Dentofacial disharmony (DFD) patients have jaw disproportions, with a high prevalence of SSDs, where the severity of malocclusion correlates with the degree of speech distortion. DFD patients often seek orthodontic and orthognathic surgical treatment, but there is limited familiarity among dental providers on the impacts of malocclusion and its correction on speech. We sought to review the interplay between craniofacial and speech development and the impacts of orthodontic and surgical treatment on speech. Shared knowledge can facilitate collaborations between dental specialists and speech pathologists for the proper diagnosis, referral, and treatment of DFD patients with speech pathologies.

## 1. Introduction

### 1.1. Significance of Speech

Speech is the primary form of language communication among humans. It consists of discrete articulatory gestures that give rise to acoustic events perceived as meaningful sounds [1]. Speech is unique to hominids, with evolutionary significance in the advancement of tools [2]. The evolution of jaw relationships with positive dental overjet has also allowed the development of labiodental fricatives including [f] and [v] [3]. Perceptually normal speech impacts how humans perceive and interact with each other. Patients with abnormal speech, especially lisping, are perceived more negatively and as intellectually inferior by peers and teachers, influencing self-confidence [4–8]. Adolescents (10–13 years old) with speech abnormalities report decreased self-esteem compared with peers [5,9]. These biases can lead to negative academic, social, and economic sequelae that persist into adulthood [6]. A longitudinal, three-decades-long study found significant differences in educational and career performance for participants with language and/or speech impairment; their high school completion rate was only 76%, compared with 92% among children without language/speech disorders [6].

### 1.2. Speech-Sound Disorders and Malocclusions

**Figure 1.**
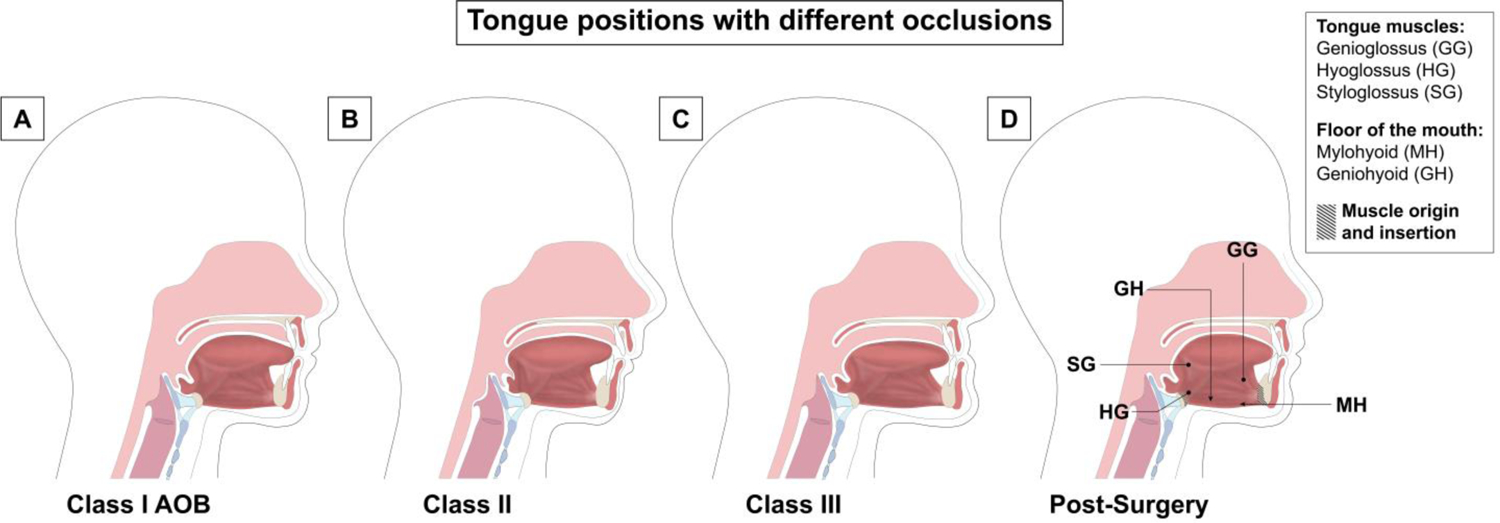
Tongue posture in DFD patients pre and postoperatively. (**A**) In DFD patients with AOB, the tongue tip is positioned more anteriorly, commonly resulting in a lisp [19–21]. (**B**) In Class II DFD patients, the back of the tongue is positioned more posteriorly in the oral cavity [22]. (**C**) In DFD patients with Class III malocclusion, the tongue appears to have a flatter surface because these patients present with a larger mandible [23]. (**D**) After orthognathic surgery, the maxilla and mandible are in a Class I skeletal relationship and anterior tooth relationships show a proper 2 mm overbite and 2 mm overjet. Tongue positioning changes postoperatively, relative to the preoperative position seen in the DFD groups. In the AOB group, the tongue placement becomes more posterior due to the positive overbite. In the Class II population, the tongue moves more anteriorly due to the mandible being advanced during surgery [22]. Postoperative DFD patients with Class III correction demonstrate increased tongue height, with a resting position against the palate and the tip of the tongue touching the lingual surface of the anterior upper incisors and the anterior palate [23]. Muscle origin and insertion points are shown by hash marks on the hyoid bone and mandible in (**D**). The genioglossus (GG), hyoglossus (HG), and styloglossus (SG) represent the lingual musculature and are indicated by lines with dots at the tip. The floor of the mouth muscles includes the mylohyoid (MH) and geniohyoid (GH), both indicated by arrows. Insertion and origin points are consistent before and after orthognathic surgery.

**Figure 2.**
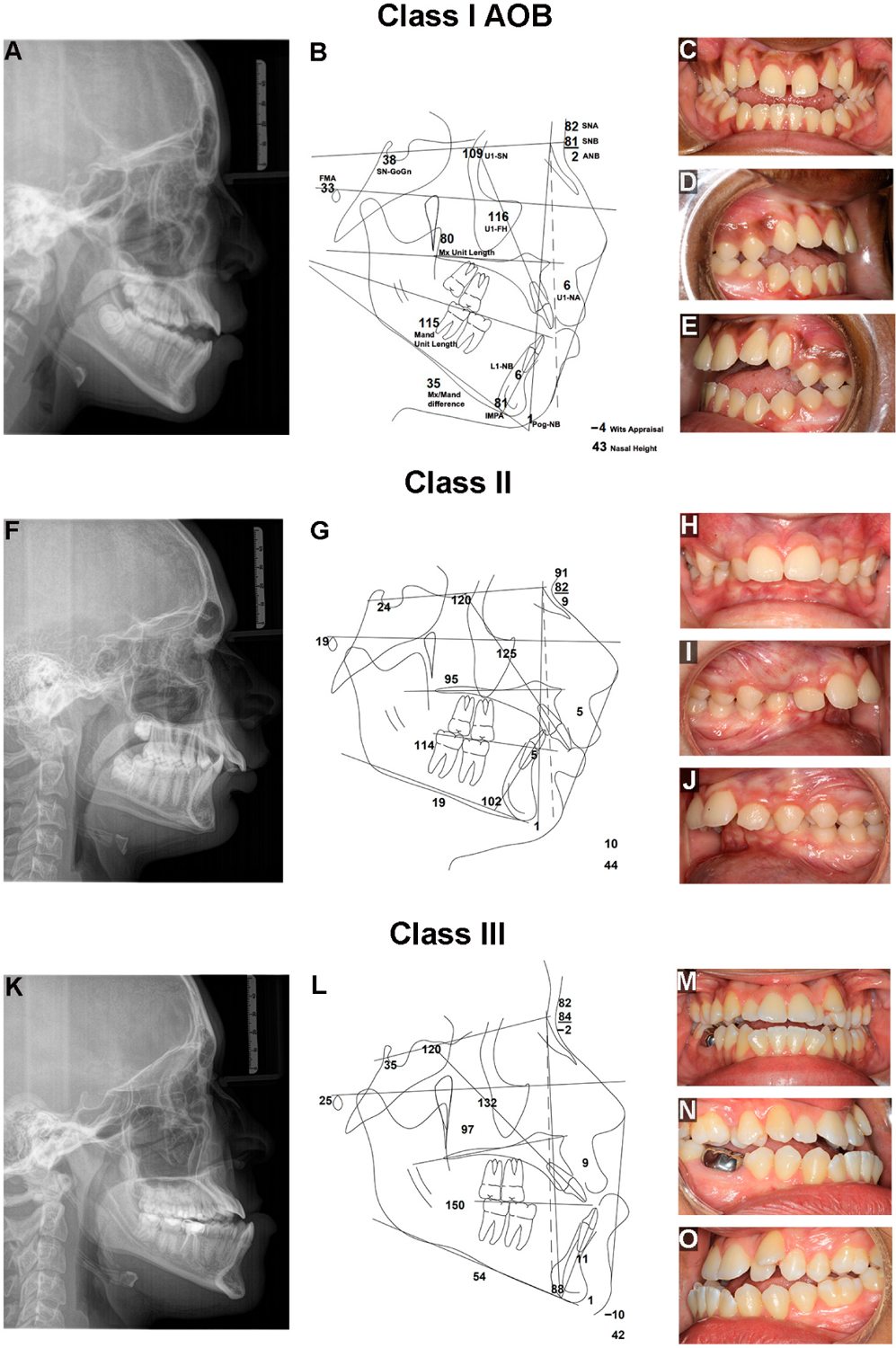
Clinical presentations of Class I AOB, II and III malocclusions in DFD patients. (**A**) Lateral cephalogram (ceph.) radiograph showing a DFD patient with a skeletal and dental AOB, with a posteriorly tipped maxillary palatal plane, a high mandibular plane angle, increased lower facial height, and two planes of occlusion in the maxillary dentition. (**B**) Ceph. tracing of a DFD patient with AOB showing references lines as well as linear (mm) and angular measurements (°), including Sella-Nasion-Gonion-Gnathion (SN-GoGn (°)), Upper 1-Sella-Nasion (U1-SN (°)), Sella-Nasion-A point (SNA (°)), Sella-Nasion-B point (SNB(°)), A point-Nasion-B point (ANB(°)), Upper 1-Nasion-A point (U1-NA (mm)), Lower 1-Nasion-B point (L1-NB (mm)), Pogonion-Nasion-B point (Pog-NB (mm)), Wits appraisal (mm), Incisor-Mandibular Plane Angle (IMPA (°)), Mandibular (Mand) Unit Length (mm), Maxillary (Mx) unit length (mm), Mx/Mand difference (mm), Frankfort Mandibular Plane Angle (FMA (°)), and Upper 1-Frankfort Horizontal (U1-FH(°)). (**C**) Frontal intraoral photo. (**D**) Right intraoral photo. (**E**) Left intraoral photo. (**F**) Lateral ceph. of a Class II, Division 1 DFD patient with excess overjet, proclined maxillary incisors, deep bite, low mandibular plane angle, and short lower face height. (**G**) Ceph. tracing of a Class II DFD patient with reference lines and measurements. (**H**) Frontal intraoral photo. (**I**) Right intraoral photo. (**J**) Left intraoral photo. (**K**) Lateral ceph. of a Class III DFD patient with mandibular prognathism, negative overjet, and proclined maxillary incisors. (**L**) Ceph. tracing of a Class III DFD patient with reference lines and measurements. (**M**) Frontal intraoral photo. (**N**) Right intraoral photo. (**O**) Left intraoral photo.

The American Speech-Language-Hearing Association (ASHA) defines speech sound disorder (SSD) as an “impairment in articulation, fluency or voice” and may include distortions, repetitive sounds, and omissions in speech sounds [10]. SSD is an umbrella term that can be linked to a number of causes, including organic and learned. Proper articulation of speech relies on precise interactions among the tongue, lips, teeth, alveolus, and jaws, which interact for speech production. These articulators are abnormally positioned in patients with a “handicapping” malocclusion or dentofacial disharmony (DFD), who make up 2.5% of the population [11]. Patients with DFD have severely aberrant skeletal and dental relationships that can interfere with mastication, temporomandibular joint function, facial esthetics, and speech [12–14]. DFD patients are commonly afflicted with articulation disorders due to abnormalities in their organic, anatomical structures. DFD patients are grouped by malocclusion including Class II (excess “overbite”, a colloquial term for overjet), Class III (underbite), and anterior open bite (AOB), as shown in Figures 1 and 2, though some patients present with both vertical and anterior–posterior (AP) discrepancies (e.g., Class III open bites). The treatment for full correction of DFD includes orthodontics and orthognathic surgery, with dental decompensation and surgical movement of the maxilla, mandible, or both into an ideal relationship, often with enhancement of facial esthetics [11]. By altering the dental and skeletal relationships in DFD patients, jaw surgery can also improve masticatory function and reduce pain in the temporomandibular joint [15,16]. Another functional benefit is improvement in respiration, particularly for patients receiving maxillomandibular advancement for sleep apnea [17]. Many patients postoperatively report improvements in sleep and general quality of life [18]. Improved speech may also be a functional benefit of jaw surgery but is still an active area of research requiring further evaluation.

### 1.3. Relationship between Speech Sound Disorders and Malocclusion

Within the general population, 4.9% of adolescents and 3.5% of adults have speech-sound disorders (SSDs) [24,25]. However, among DFD patients, a noTable 90% of Class III patients, 83% of AOB patients, and 73–87% of Class II patients present with SSDs [4,26]. Patients with DFD present with a biological or organic impediment to proper speech, akin to a motor speech disorder. However, SSDs in DFD patients result not from a neurological or muscular pathology but from structural abnormalities in their vocal tract, particularly within the oral cavity. As a result, we describe patients with DFD as having structural SSDs, with obligate speech errors and distortions due to abnormal oral and vocal tract anatomy.

The large discrepancy in prevalence of articulation pathologies between DFD subjects and the general population suggests a direct relationship between speech distortion and severe malocclusion. The interaction between speech, malocclusion, and its correction is an area of active investigation, with implications for the clinical management of DFD patients with SSDs [26]. Understanding the principles of speech pathology and its presentation and management in DFD patients is relevant for speech-language pathologists (SLPs), orthodontists, and oral and maxillofacial surgeons (OMFSs). OMFS and orthodontic providers manage care for DFD patients over years and are uniquely positioned to evaluate malocclusion severity and detect speech issues for proper referral and interdisciplinary management with SLPs early in development.

We aimed to review the key stages of speech and craniofacial development as a foundation for understanding how DFD and its correction influence speech. Furthermore, we aimed to describe the differences in speech distortions among Class II, Class III, and anterior open bite DFD patients and review the current literature on postoperative speech in these patient groups.

## 2. Speech and Craniofacial Development

### 2.1. Development from Birth to Seven Months Old

**Figure 3.**
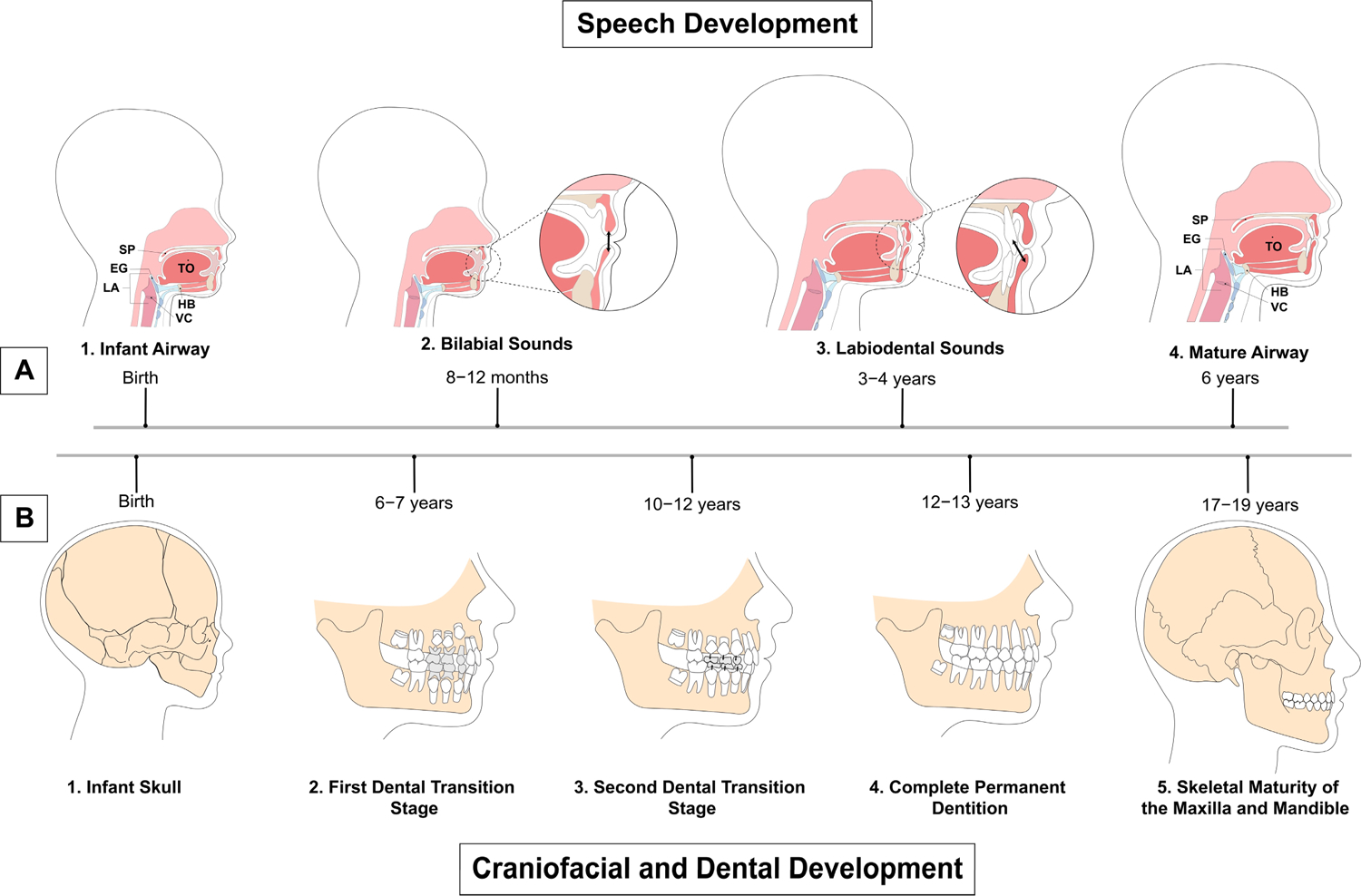
Timeline of speech, craniofacial, and dental development. (**A**) Speech development from birth to maturity. 1. Airway structures in a child at birth. 2. At 8 to 12 months, the child produces primarily bilabial sounds with the absence of teeth. 3. Labiodental sounds start to be produced at 3 to 4 years old with the eruption of teeth, similar to the adult stage. 4. Maturity of the airway at 6 years old. Structures are consistent through adulthood. (**B**) Craniofacial and dental development from infancy to adulthood. 1. Infant skull at birth. 2. First transition stage of dental development with eruption of first permanent molars, and central and lateral incisors at 6 to 7 years old, establishing the mixed dentition. The first transition stage is followed by the interphase dental period from 8 to 9 years old, when the mixed dentition remains stable. 3. The second dental transition stage from 10 to 12 years old includes exfoliation of all remaining primary teeth, with replacement of deciduous canines and premolars with permanent successors. 4. Complete permanent dentition at 12 to 13 years old. 5. Skeletal maturity of the maxilla and mandible at 17 to 19 years old. SP: soft palate; EG: epiglottis; LA: larynx; TO: tongue; HB: hyoid bone; VC: vocal cords.

Speech and the craniofacial complex simultaneously develop during childhood (Figure 3). Immediately after birth, vocal development begins as a baby takes its first cry. At birth, the mandible is underdeveloped with a short ramus (Class II profile), the larynx is high in the throat, and the tongue takes up much of the oral cavity space [27,28]. The alveolar ridges develop along with the underlying tooth buds, and gum pads eventually begin to touch [28]. Anatomical changes in the jaws, tongue, alveolus, and vocal tract affect the types of sounds infants are able to produce.

The stages of infant sound development include phonation (i.e., crying), the goo/coo stage, expansion, reduplicated and no-reduplicated babbling, and the one-word and then the two-word stages [27]. From birth to one month of age, an infant communicates through crying, which consists of vowel sounds such as “oohs” and “aahs” during the phonation stage. At two months, the infant enters the cooing period and begins to develop front and central vowels along with back consonants such as [k], hence the name of the stage. At around four months of age, the larynx, hyoid, and tongue descend, and the laryngopharynx lengthens, allowing for a wider range of sound production including isolated vowels and the beginning of other consonants [29]. The expansion stage (four to seven months) is initiated when the mandible undergoes downward and forward growth, increasing oral space for vowel production [27].

### 2.2. Development from Seven to Twelve Months Old

The babbling period occurs from seven to twelve months and begins with infants imitating speech and understanding word meaning. Often, their first word stems from babbling and imitation (such as ma-ma-ma), which is then reinforced by those around them repeating the word mama. From eight to twelve months, oral stops [b, p] and nasals ([m] mama and [n] no) are the most frequently produced consonants. Sounds produced in babbling are typically acquired in order of ease of production. Bilabials (e.g., [b] baba) tend to be amongst the first sounds produced due to their strong visual cues. Stops (e.g., [p] pea), nasals, and glides (e.g., [j] yes and [w] why) also dominate speech in the early periods, while fricatives (e.g., [s] see), affricates (e.g., [tʃ] cheer), and liquids (e.g., [l] led, [ɹ] red) appear later, suggesting greater motor control is required for these sounds. At the end of the babbling period, children use approximately two times as many vowels as consonants and begin to develop stops and fricatives [26]. Stops, such as the /p/ sound, are made when complete closure of the vocal tract prevents air from escaping, while fricatives such as [f] involve air escaping through a small constriction producing turbulent airflow [30].

### 2.3. Development from One to Three Years Old

Speech development, during and after the babbling period, is influenced by the eruption of primary teeth and the development of the supporting bony alveolus. At around six months of age, the primary central and lateral incisors begin to erupt, when infants are ready to be weaned, and by twenty-three to thirty-three months of age, babies have a full complement of primary dentition (twenty primary teeth in total). A concomitant increase in vertical facial height yields space between the gum pads to accommodate the erupting primary teeth [11]. The alveolus and dentition significantly develop and can assume their roles as passive articulators for the tongue and lips to act against as active articulators. At one to two years of age, children acquire nearly as many consonants as vowels and begin to learn both dental and postdental sounds such as [f] and [d] [31]. As this occurs, deciduous (i.e., primary) canines and first molars begin to erupt into the oral cavity, while deciduous incisors have fully erupted [32].

### 2.4. Development from Three to Five Years Old

Two to four months after children’s first word, they begin their two-word stage, forming short telegraphic sentences (such as mama go). Typically, the two-word stage begins following the acquisition of roughly forty words [33]. Entering the third year, children can recursively combine phrases, allowing multiword phrases, and children learn to produce all vowels and around two-thirds of consonants and liquids (liquids are vowel-like consonants such as the [l] in led and the [ɹ] in red) [34]. Children learn they can communicate through speech: crying when sad or complaining when angry motivates actions from those around them. Dentally, second primary molars erupt to complete the primary dentition between twenty-three and thirty-three months [32]. During early years, non-nutritive sucking habits are common (i.e., thumb sucking) and are seen in 73% of children at ages two to five [35]. Sucking habits are associated with anterior open bites and posterior crossbites, impacting speech, and need to resolve by four to six years old for self-correction to occur without orthodontic intervention.

As children continue to develop, their oral functions become refined, including breathing, sucking, swallowing, and chewing [36]. By the age of four, children typically know 77% of sounds; knowledge of sounds continues expanding until the age of seven or eight. By four, the mandible will have increased in length and ramus height, leading to a decreased gonial angle; by seven, the cranial base will have lengthened, moving the maxilla and mandible downward and forward [28,37]. The pattern of downward and forward facial growth continues throughout craniofacial development [28]. The maxilla and mandible develop as modeled by Scammon’s growth curves, with the maxilla developing earlier (similar to the neural curve) and the mandible growing longer and later (similar to the somatic general body curve) [28]. The maxilla undergoes anterior surface remodeling (i.e., resorption) and growth via bone apposition (i.e., deposition) at its superior and posterior sutures, displacing the maxilla downward and forward and increasing the oral cavity size; bony deposition at the posterior aspect of the alveolar processes creates more space for distal molar tooth eruption [28,38]. Mandibular growth occurs later than maxillary development, up through 18–20 years of age, and includes downward and forward displacement, with the condyle growing upward and backward; the posterior ramus undergoes appositional growth, and the anterior ramus resorbs, increasing space for distal molar tooth eruption [28,39]. Oral cavity size increases, with increasing facial dimensions in the transverse, anterior–posterior, and vertical dimensions [40,41]. The vocal tract also lengthens, lowering its resonant frequencies [42].

### 2.5. Development from Six to Nine Years Old

From age six to seven, the permanent first molars erupt distal to the primary second molars, lengthening the dental arch; additionally, deciduous central incisors and then lateral incisors are exfoliated with the eruption of their permanent successors as part of the first transition stage of dental development [32] (Figure 3). The interphase dental period then begins from ages eight to nine (i.e., the intermediate intertransitional phase), which is the one to two-year span when the mixed dentition remains relatively stable, with permanent incisors and first molars [43]. If articulation errors exist at age eight to nine, self-correction is unlikely, and intervention may be needed. By eight years old, children typically have mastered the sounds of their primary language [31]. Generally, early speech deficits are referred to a SLP; however, due to the mild nature of most deficits, most children are not referred until school age, at which time they may be diagnosed with an SSD [26]. An articulation disorder is a type of SSD characterized by “atypical production of speech sounds characterized by substitutions, omissions, additions or distortions that may interfere with intelligibility” [10]. Articulation disorders are often seen in DFD patients with severe malocclusions and jaw disproportions [19,26,44]. Starting at age seven, the American Association of Orthodontics (AAO) recommends that children be referred to an orthodontist for an initial evaluation [45]. As a result, orthodontists can play an important role in diagnosing significant malocclusions and speech abnormalities, and appropriately referring to SLPs for treatment in conjunction with Phase I and II orthodontic care.

### 2.6. Development from Ten to Twelve Years Old

The second dental transition stage occurs from ages ten to twelve years old and includes the replacement of deciduous canines and molars with permanent canines and premolars and a slight reduction in dental arch length [46]. By puberty, when most patients are undergoing comprehensive Phase II orthodontic treatment and vocal tracts are lengthening, speech development has concluded, with few corrections, if any, occurring without intervention [2]. Speech sound acquisition is judged to be complete, with adult-like motor control, at about eleven or twelve years old [47]. It is interesting to note that speech errors are engrained by the interphase period of dental development (ages eight to nine) and that speech development fully plateaus when the permanent dentition is in place at puberty. Speech, craniofacial, and dental development occur in parallel with one another, with each developmental process influencing the other.

## 3. Effects of Malocclusion on Speech

Severe ‘handicapping’ malocclusions are associated with a 16- to 25-fold increased prevalence of speech distortions compared with Class I controls [48–50]. DFD subtypes (e.g., Class II, Class III and AOB) demonstrate distinct features on acoustic and perceptual evaluations. This suggests that vertical and anterior–posterior disproportions have separate and potentially additive impacts on articulation [48–50]. Among DFD patients, 90% of Class III and 83% of open-bite patients suffer from SSDs compared with 4.9% of adolescents and 3.5% of adults in the general population, suggesting a link between jaw disproportion and articulation issues [24]. Similarly, Vallino et al. found 88% of patients with severe malocclusions exhibited articulation errors [48]. DFD patients often list articulation over other factors such as chewing as a motivation for seeking surgical orthodontics [51]. Severe malocclusion has been implicated as a cause of speech distortion, especially in the pronunciation of sibilants [s] and [z] [44–46]. Linear correlations have been found between the severity of malocclusion and the degree of distortion, suggesting causation between malocclusion and speech distortions [19,26,44].

Each DFD group exhibits different oral anatomies and different speech presentations, as described below and in Table 1. Table 2 defines methods of how speech has been studied in DFD groups, and Table 3 provides a glossary of distortion types, with a focus on lisping, frequently seen in DFD patients. For these tables and this review, we conducted a literature search within the PubMed and Google Scholar databases in January 2023. The main keywords used during the search were “speech and malocclusions,” “orthognathic surgery,” “dentofacial disharmonies,” “dentofacial deformities,” “speech distortions and anterior open bite,” “speech distortions and Class III,” and “speech distortions and Class II.” We extended our search to the bibliographies of relevant, selected publications. Only English full-text manuscripts published in peer-reviewed journals were included in the search. We excluded studies primarily focusing on maxillomandibular advancement as well as cleft lip and palate procedures, and other nonorthognathic oral surgeries. Table 1 presents the major results of our literature search.

### 3.1. Vertical Discrepancies: Anterior Open Bite

An anterior open bite (AOB) exists when there is negative overbite, where maxillary teeth fail to overlap the mandibular teeth. DFD patients with AOB commonly have difficulty incising food, abnormal tongue posture, esthetic concerns, and aberrant speech. AOB is the most common malocclusion associated with SSDs, with 75–83% of AOB patients diagnosed with speech distortions [19]. Several studies noted that patients with AOB show increased rates of sound production errors, with interdental lisping as a common visual and auditory distortion [19–21]. Common SSDs in the AOB population also include sibilants such as [s] and [z] as well as postalveolar affricate [tʃ] (<ch>) and labiodental fricatives [f] and [v] [73,74]. Keyser et al. found a 10-fold increased prevalence of interdental and auditory distortions compared with controls for sequences including /ta/, /la/, /sa/, /si/, and /sIsi/ [19,66]. Similarly, sibilants [s] and [z] were aberrant in several studies of AOB patients, with one study identifying articulation problems in 84.4% of children with AOB [61]. Linear correlations were also found between open-bite severity and degree of speech distortions [19].

### 3.2. Anterior-Posterior Discrepancies

*Class III*: Speech distortions are well-documented in the Class III population [44,50]. A study of 451 Finnish students found that those with Class III malocclusions were 4.5 times more likely to produce consonants more anteriorly [66]. In our study of 102 patients with Class III malocclusions, we observed perceptual distortions of /sa/, /si/, and /sIsi/ in 63.73%, 61.11%, and 55.56% of Class III patients compared with 1.61%, 2.50%, and 2.50% of controls, respectively [26]. The severity of Class III malocclusion is also linearly correlated with the degree of speech distortion, specifically for sounds [t] and [k] [26]. Fricatives such as [f], [s], and [z] are frequently affected in Class III patients due to either a prognathic mandible, a retrognathic maxilla or a combination of both; SSDs are found 20 times more often in the Class III population than in the general population [49,75].

The Class III skeletal discrepancy may lead to speech distortions due to alterations in the structure of the anterior oral cavity, where many consonant sounds are articulated [3,4,20,26,76]. Class III underbites are characterized by mandibular incisors being positioned anteriorly of their maxillary incisors, influencing the articulation of alveolar sibilant fricatives ([s] and [z]), where the tongue normally interacts with the maxillary alveolus, and labiodental fricatives ([f] and [v]), where the lower lip typically meets the maxillary incisors. To adjust for an underbite, Class III patients produce sounds with compensatory articulation gestures, such as the upper lip contacting the mandibular incisors to produce fricatives [f] and [v], and the tongue contacting the incisors instead of the alveolar ridge for sibilants [s] and [z] [75]. However, this compensation is often inadequate, leading to increased incidence of speech distortions [48,57].

*Class II*: Class II malocclusions have been linked to distorted speech, but Class II DFD patients have received less attention in the speech literature, despite Class II malocclusions constituting a majority of orthodontic patients [44,77]. This dearth of studies may be due to the smaller spectral shifts associated with fewer sounds among Class II patients compared with other DFD cohorts [44]. Class II patients can temporarily posture their lower jaw anteriorly into a normal Class I position (“Sunday bite”): a compensatory movement that is not possible with Class III and AOB DFD groups (as patients cannot voluntarily move their maxilla or retract their mandible without surgery) [78]. Compensation may help Class II patients approximate normal [s] and [z] articulation [48]. In some cases, attempting compensatory articulation contributes to distortions, with the protrusion of the tongue past the incisors (“interdental lisping”) during the production of /s/, leading to an audible lisp [48,50]. Perceptually, this imprecise articulation of sibilants [s] and [z] can be attributed to a reduced oral opening caused by the deep bite seen in many Class II patients [72,75]. Both our study at UNC and a Turkish study applied spectral moment analysis and found differences in [s] articulation in Class II patients compared with controls, indicating speech distortions are present at an increased prevalence among Class II patients, despite attempted compensation [44,50]. Though Class II DFD patients have documented articulation issues, their speech presentation may be milder than that in other DFD groups, with smaller shifts in consonant spectral moments (centroid frequency (M1) and spectral spread (M2)), compared to in AOB and Class III DFD cohorts [44]. Additionally, bilabials can sound distorted in Class II patients as the lower lip contacts the upper incisors rather than the upper lip due to excessive overjet [72,75]. One study found distortion of bilabial sounds was unique to patients with Class II malocclusions compared with those with Class III bites [72]. The characterization of speech distortions and severity requires greater investigation among Class II DFD patients to understand the spectrum of presentations and influence of compensation.

## 4. Effects of Orthognathic Surgery on Speech

For full correction, Class II, Class III and AOB DFD malocclusions are treated with a combination of orthodontics and orthognathic jaw surgery. Preoperative studies have demonstrated that speech distortions of DFD patients are related to their skeletal discrepancies and that malocclusion severity correlates with the degree of speech distortions (Table 1) [19,26,44]. However, the influence of surgical correction on speech is an area of active inquiry, with conflicting results and diverse sample sizes (Table 1). Comparing these studies is difficult due to the differences in the language evaluated, length of postoperative follow up (Table 1), methods used (Table 2), and use of a control population. Despite these challenges, many postoperative studies have indicated promising improvements in speech, with either an elimination or reduction in articulation errors regardless of the type of preoperative malocclusion [62]. While some studies involved small samples, they suggest an important trend in surgery alleviating speech errors in some DFD cohorts. A synthesis of postoperative speech outcomes for each DFD group is discussed below, and current literature is summarized in Table 1.

### 4.1. Vertical Discrepancies: Anterior Open Bite

The articulation errors and postoperative changes observed in AOB DFD patients are highly variable across studies. According to Vallino et al. (N = 17 AOB), the reduction in errors produced by AOB patients is minimal at 3 months postoperation [69]. Similarly, Knez et al. found no significant differences in speech 6 months postoperation in patients with AOB (N = 15) [21]. In contrast, Turvey et al. found 88% of AOB subjects (N = 9) exhibited positive changes in speech at 12 months after surgery [62]. The few studies specific to AOB lacked sufficient sample sizes and control cohorts for comparison, preventing definitive conclusions on the impact of jaw surgery on speech of AOB patients.

### 4.2. Anterior–Posterior Discrepancies

Class III: Studies have indicated Class III patients have variable speech outcomes following orthognathic surgery, with most studies using perceptual evaluations of recordings (Table 2). Goodstein et al. (N = 10) found no significant changes in speech patterns at 2 months postoperation, when patients are still quite swollen; others noted that some patients had reduced frequencies of speech errors postoperatively at 2-, 3-, and 6-month time points [52–55,68]. One study of Class III patients (N = 20) that underwent two-jaw surgery found a decrease in speech errors of consonants [s], [ʃ], [z], and [ɹ] with complete elimination of errors by 6 months postsurgery in all subjects [54]. Similarly, Ruscello et al. (N = 20) found 41.7% of Class III subjects experienced correction of speech errors at 6-months postoperation [68]. A small, underpowered study (N = 4), by Bruce and Hanson, showed improved articulation in two of their participants [53]. Most studies have indicated qualitative improvement in speech in 41–100% of Class III patients. Similar to AOB studies, manuscripts on Class III DFD subjects have small sample sizes and lack comparison with Class I control groups, preventing definitive conclusions.

Class II: In a study by Niemi et al., Class II DFD patients (N = 5) with no significant speech distortions were evaluated up to 30 weeks postoperatively [60]. Postoperative changes were highly variable across individuals. For example, one patient demonstrated a decrease in F1 values for vowels /æ, a/, and another patient exhibited decreases in F1 for all vowels, except mid front vowels /e, ø/. Vowel sounds can be produced without articulation with skeletal structures (such as the tongue with the palate), making interpretation challenging; vowel sound production in Class II patients can be varied due to compensations in tongue or jaw positions and possible differences in vocal tract anatomy [60].

Literature focused on Class II DFD patients’ postoperative outcomes is quite sparse. Instead, most studies evaluating speech outcomes have utilized an unstratified mix of DFD patients with markedly different occlusions: this is a methodological issue in need of careful consideration. In a study of 12 DFD patients who produced preoperative articulation errors, 5 subjects (n = 2 Class II, 3 Class III) exhibited complete correction of their speech errors after surgery and another 5 had a decrease in the number of errors (n = 1 Class II; 3 Class III; 1 facial asymmetry) [68]. Ward et al. noted modest changes in some DFD patients’ speech after surgery (n = 5, one Class III, three Class II and one Class III/AOB); these slight changes in a small, heterogeneous sample are encouraging but make it difficult to draw definitive conclusions [71]. Another study of 41 patients with varying preoperative malocclusions (n = 29 Class II, 11 Class III, and 25 AOB) found a significant improvement in speech on 70 test sounds, 6 months after surgery. While some DFD groups improved less than others, all experienced a reduction in speech errors [57]. The variability in results likely stems from small sample sizes, heterogenous DFD cohorts (Classes II, Class III and AOB), and use of qualitative methods (i.e., perceptual visual and auditory analysis), exemplifying the need for additional research, with data stratified by DFD group and sufficient power.

## 5. Final Considerations

The functional benefits of orthognathic surgery may include improvements in mastication, respiration, sleep, temporomandibular joint dysfunction, quality of life, and self-esteem. The extent of these benefits may vary by malocclusion and by patient. It is well-supported in the literature that patients with DFD present with a significantly higher incidence of speech distortions than patients with Class I occlusion. However, speech, as a functional benefit of orthognathic surgery, is an area of active investigation and much is still unknown. Postoperative speech outcomes are ambiguous following DFD correction, with significant variability across studies, particularly for Class II and AOB DFD cohorts. Most Class III postoperative studies show some degree of improvement, suggesting that surgical correction influences speech. While most studies suggest positive change in speech, the extent and duration of improvement are unknown. Well-controlled, longitudinal studies with adequate sample sizes, quantitative measures, and data stratification by malocclusion group are needed to determine if speech improves postoperatively in DFD subjects and to provide evidence-based recommendations for the clinical management of DFD patients with speech concerns.

Speech and occlusion develop in parallel throughout childhood, with each process likely influencing the other. In growing children, where Phase I interceptive orthodontics may correct discrepancies, a combination of orthodontics and speech therapy may simultaneously resolve both the malocclusion and speech issues. In nongrowing young adults, orthognathic surgery with orthodontics and postoperative speech therapy may be required to correct obligate distortions and the malocclusion. Interdisciplinary management of nongrowing DFD patients by orthodontists, SLPs, and oral surgeons may be necessary for speech improvement following malocclusion correction. Having a working knowledge of speech pathology associated with DFDs will allow providers to screen for articulation errors, answer questions, and guide appropriate interdisciplinary referral and care at all ages. This may represent an opportunity for dental providers to expand our impact in overall health, quality of life, development, and function.

## Figures and Tables

**Table 1.** Speech evaluations and results by malocclusion type.

Author, Year, and Language	Sample Size, DFD Groups and Ages	Methods Used	Preoperative Findings	Timepoints Evaluated	Postoperative Findings
DFD Group: Class III					
Ahn et al., 2015 [52] Language: Korean	N = 16 Class III n = 8 Controls* n = 8 Ages 18–26	Formant analysis of vowels	Class III DFD patients used an area of the formant graph 133.44% larger than the controls.	Presurgery, 6 weeks, 3 and 6 months postsurgery	F1 and F2 formants of Class III patients reduced in vowels [a], [i], [e], and [æ] by 6 months postsurgery; articulating positions shifted.
Bruce and Hanson, 1987 [53] Language: English	N = 4 Class III n = 3 Asymmetry n = 1 No controls Ages 16–43	Perceptual evaluation of recordings; tongue thrust evaluation	100% of DFD patients presented with a lisp and tongue thrust. Distortions were seen in [s] and [z] sounds.	Presurgery, 9 weeks postsurgery	Correction of speech in 50% of patients, 25% of those with tongue thrust improved.
Ghaemi et al., 2021 [54] Language: Persian	N = 20 Class III n = 20 No controls Ages 18–40	Perceptual evaluation of recordings; formant analysis of vowels	95% of Class III DFD patients produced distortions in consonant sounds [ʃ] and [s]. Distortions were also present in [ɹ] and [z] sounds in the majority of participants.	Presurgery, 1 and 6 months postsurgery	All articulation errors eliminated by 6 months postoperatively; speech intelligibility increased to 100% at 6 months postsurgery.
Glass et al., 1977 [55] Language: English	N = 5 Class III n = 5 No controls Ages 18–54	Perceptual evaluation of recordings	All Class III DFD patients had speech articulation distortions.	Presurgery, 2 months postsurgery	Decrease in sibilant distortions in 100% of patients.
Goodstein et al., 1974 [56] Language: English	N = 10 Class III n = 5 Controls n = 5 Ages: N/A	Perceptual evaluation of recordings	100% of Class III DFD patients had preoperative speech errors.	Presurgery, splint removal, 2 months postsurgery	More fluent speech postoperatively, but no significant changes in speech pattern in 100% of Class III patients.
Guay et al., 1978 [57] Language: English	N = 12 Class III n = 12 No controls Mean age: 13	Live perceptual evaluation; cephalometric analysis	92% of Class III patients had some degree of distortion of [s]. Tongue posture at rest was lower than normal.	Presurgery	NA: Postoperative outcomes were not studied.
Lathrop-Marshall et al., 2022 [26] Language: English	N = 164 Class III n = 102 Controls n = 62 Ages 14–40	Perceptual evaluation of recordings; cephalometric analysis; spectral moment analysis	Severity of malocclusion correlated with distortion of [t] and [tʃ] in Class III patients.	Presurgery	NA: Postoperative outcomes were not studied.
Weimer and Astrand, 1977 [58] Language: Swedish	N = 30 Class III n = 30 No controls Ages 18–45	Perceptual evaluation of recordings	Mild speech defects were seen in 17% of Class III DFD patients preoperatively; 83% of the patients were considered to have normal speech.	Presurgery, 6 months postsurgery	60% of patients with preoperative speech defects had correction in speech, 40% of those patients experienced slight improvement in speech.
DFD Group: Class II					
Garber et al., 1981 [59] Language: English	N = 6 Class II n = 6 No controls Ages 14–24	Perceptual evaluation of recordings; cephalometric analysis	Average of 35 errors in speech were noted during the presurgical recordings among Class II DFD patients.	Presurgery, 5 days; 1, 3, 6, and 12 months postsurgery	Speech deterioration was noted immediately after surgery, predominantly in phoneme [s]; there was overall improvement long-term after surgery.
Niemi et al., 2006 [60] Language: Finnish	N = 5 Class II n = 5 No controls Ages 31–42	Formant analysis of vowels; cephalometric analysis	None of the subjects had speech disorders or difficulties despite having DFD.	Presurgery, 6 and 30 weeks postsurgery	No significant long-lasting changes were found postoperatively.
DFD Group: Anterior Open Bite (AOB)					
Keyser et al., 2022 [19] Language: English	N = 101 AOB n = 39 Controls n = 62 Ages 14–40	Perceptual evaluation of recordings; spectral moment analysis; cephalometric analysis	Higher prevalence of distorted [s] found in AOB patients.	Presurgery	NA: Postoperative outcomes were not studied.
Knez Amrožič et al., 2015 [21] Language: Slovenian	N = 15 AOB n = 15 No controls Ages 18–32	Formant analysis of vowels; cephalometric analysis	60% of AOB DFD patients had articulation disorders.	Presurgery, 6 months postsurgery	No significant changes were found postoperatively.
Kravanja et al., 2018 [61] Language: Slovenian	N = 75 AOB n = 32 Controls n = 43 Ages 3–7	Live perceptual evaluation; ultrasound imaging of tongue	84% of AOB patients had articulation disorders and 81% of AOB patients had abnormal tongue posture.	Presurgery	NA: Postoperative outcomes were not studied.
Turvey et al., 1976 [62] Language: English	N = 9 Class III/AOB n = 2 Class II/AOB n = 4 Class I/AOB n = 3 No controls Ages 14–27	Live perceptual evaluation; cephalometric analysis; tongue thrust evaluation	89% of DFD patients presented with perceptible lisping preoperatively.	Presurgery, 3-, 6- and 12-months postsurgery	78% had improvement in lisping; all patients improved in tongue function.
DFD Group: Multiple Malocclusions					
Bowers et al., 1985 [63] Language: English	N = 5 Class III n = 2 Class II n = 3 No controls Ages 17–22	Perceptual evaluation of recordings; formant analysis of vowels	All patients had perceptually normal speech preoperatively.	Preorthodontic treatment, presurgery, postsurgery, postdebonding	Significant frequency shift for [e]; speech was perceptually normal postoperatively.
Buyuknacar et al., 1993 [50] Language: Turkish	N = 60 Class III n = 20 Class II n = 20 Controls n = 20 Mean age: 14	Spectral moment analysis; cephalometric analysis	Center of gravity for [s] was lower in Class II patients compared with others. No evidence for correlation between malocclusion and speech disorder.	Presurgery	NA: Postoperative outcomes were not studied.
Dalston and Vig, 1984 [64] Language: English	N = 40 Class III n = 25 Class II n = 15 No controls Ages N/A (adults)	Perceptual evaluation of recordings; velopharyngeal evaluation; cephalometric analysis	More than half of the errors were made by 20% of all patients. Most of the errors were distortions of [s] and [z].	Presurgery, 6 and 12 months postsurgery	Nasal–oral coupling and nasal resistance significantly improved; no significant perceptual changes in speech postoperatively.
Geffen, 1978 [65] Languages: English and Afrikaans	N = 9 Class III n = 6 Class II n = 2 Asymmetry n = 1 No controls Ages N/A (adults)	Perceptual evaluation of recordings; cephalometric analysis	67% of Class III DFD patients had distortions of [s]. All Class II and asymmetric patients had distortions of the [s] sound.	Presurgery, 3–11 months postsurgery	22% had improvement in articulation of [s] phoneme; 55% had improvement in general quality of speech; articulating positions shifted.
Laine, 1992 [66] Language: Finnish	N = 451** Class III n = 25 Class II n = 70 AOB n = 40 Controls n = 90 Other n = 226 Mean age: 23	Perceptual evaluation of recordings	53% of Class III, Class II and AOB patients had speech disorders; most common disorders being those produced anterior to the correct location of articulation.	Presurgery	NA: Postoperative outcomes were not studied.
Leavy et al., 2016 [20] Language: English	N = 115 Class III n = 8 Class II n = 47 AOB n = 31 Controls n = 60 Ages 8–36	Perceptual evaluation of recordings	62% of all subjects (with or without malocclusions) had articulatory distortions, mainly of [s] and [t] sounds; more severe malocclusion, more likely to have a speech distortion.	Presurgery	NA: Postoperative outcomes were not studied.
Lichnowska et al., 2021 [67] Language: Polish	N = 37 Class III n = 28 Class II n = 9 No controls Ages 18–50	Perceptual evaluation of recordings; tongue thrust evaluation	100% of patients presented with articulation concerns (by inclusions criteria); distortions in Class III patients were worse than in Class II.	Presurgery	NA: Postoperative outcomes were not studied.
Oliver et al., 2022 [44] Language: English	N = 227 Class III n = 102 Class II n = 53 Controls n = 72 Ages 12–37	Perceptual evaluation of recordings; spectral moment analysis	Greater occurrence of distortions among Class II DFD patients compared with controls; lower consonant spectral moments for Class II compared with Class III and AOB DFD patients.	Presurgery	NA: Postoperative outcomes were not studied.
Ruscello, 1986 [68] Language: English	N = 20 Class III n = 11 Class II n = 3 Asymmetry n = 2 Maxillary Excess n = 4 No controls Ages 17–53	Perceptual evaluation of recordings	About 60% of all DFD patients exhibited preoperative articulation errors.	Presurgery, splint removal, 3 and 6 months postsurgery	42% (of those with errors prior to surgery) showed reduction in errors postoperatively; 17% remained unchanged.
Vallino, 1990 [69] Language: English	N = 34 Class III n = 11 Class III/AOB n = 5 Class II n = 23 Class II/AOB n = 12 No controls Ages 14–48	Live perceptual evaluation; velopharyngeal evaluation	88% of all DFD patients showed articulation errors with distortions of sibilants [s] and [z] being the most commonly observed.	Presurgery, 3, 6, 9, and 12 months postsurgery	57% (of those with errors prior to surgery) experienced correction of speech; 43% improved; surgery did not impact velopharyngeal area.
Vallino et al., 1993 [48] Language: English	N = 33 Class III n = 6 Class III/AOB n = 4 Class II n = 12 Class II/AOB n = 11 No controls Ages 14–39	Live perceptual evaluation; cephalometric analysis	88% of all patients had articulatory distortions; most of them associated with sibilant sounds [s] and [z].	Presurgery	NA: Postoperative outcomes were not studied.
Wakumoto et al., 1996 [70] Language: Japanese	N = 5 Class III n = 3 Class II n = 2 No controls Ages 17–31	Electropalatography; spectral peak analysis	None of the patients had preoperative speech disorders when judged by an SLP.	Preorthodontic treatment, presurgery, 3 and 6 months postsurgery	Articulating positions shifted for 100% of patients; significant acoustic changes in 40% of patients.
Ward et al., 2002 [71] Language: English	N = 13 Class III n = 1 Class III/AOB n = 1 Class II n = 3 Controls n = 8 Ages 15–21	Perceptual evaluation of recordings; velopharyngeal evaluation	80% of Class III, Class III/AOB and Class II DFD patients had articulatory distortions of lingual alveolar and palatal sibilants.	Presurgery, 6 months postsurgery	25% (of those with errors prior to surgery) improved in articulation; 60% had improved interlabial pressures.
Witzel et al., 1980 [72] Language: English	N = 41 Class III n = 4 Class III/AOB n = 7 Class II n = 12 Class II/AOB n = 17 AOB n = 1 No controls Ages 9–26	Live perceptual evaluation	54% of DFD patients showed articulation errors. All groups had distortions of sibilants (except the patient with apertognathia). Labiodental distortions were noted in Class III patients. Bilabial sound distortions were noted in Class II patients.	Presurgery, 6 months postsurgery	64% (of those with errors prior to surgery) saw correction of speech; 36% of those saw improvement in speech.

* Control = Class I, no AOB.

** Occlusal classifications reported.

**Table 2.** Speech analyses used in DFD studies*.

Analysis Type	Analysis	Description
Perceptual	Live perceptual evaluation	Real time visual/perceptual evaluation by a speech pathologist at scheduled time intervals.
Perceptual	Perceptual evaluation of recordings	Visual/perceptual evaluation from a video/audio recording by a speech pathologist at scheduled time intervals.
Acoustic	Cephalometric analysis	Utilizing lateral cephalometric radiographs to analyze bony and soft tissue landmarks to relate the cranial base, maxilla, and mandible to the teeth.
Acoustic	Electropalatography	Utilization of a palatal stent with electrodes to record tongue and palate contacts during speech.
Acoustic	Formant analysis of vowels	A method used to analyze vowel pronunciation. The first formant (F1) and the second formant (F2) are typically extracted from a speech recording. An F1xF2 vowel plot is then used to display vowel sound distribution.
Acoustic	Spectral moment analysis	A type of spectral analysis typically used to describe consonants. The power spectrum is treated as if it is a probability distribution.
Acoustic	Spectral peak analysis	A type of spectral analysis typically used to describe consonants. Spectral peaks are measured.
Acoustic	Tongue thrust evaluation	Visual analysis of tongue position during different actions. Tongue position is ranked on a subjective, predetermined scale.
Acoustic	Velopharyngeal evaluation	Estimation of the size of velopharyngeal port area using pressure–flow measurements while participants are asked to repeat pressure sounds, such as [p].

* Includes speech analysis methods used in DFD studies. This is not an exhaustive list of all available speech analyses.

**Table 3.** Glossary of terms describing articulation errors associated with DFD.

Distortion	Description
Auditory distortion	Sound produced is perceived as aberrant but may look acceptable to the listener.
Visual distortion	Sound is perceived as correct but looks abnormal to the listener. Example: Speaker who produces a bilabial sound /p,b,m/ by placing the lower lip against the upper incisors. Consonant sounds normal but looks incorrect.
Lisp	A type of functional speech disorder: usually a phonetic disorder, meaning the affected person struggles to correctly position the tongue, lips, teeth, and jaw to achieve the attempted sound. Lisps are the most commonly identified and widely recognized speech problem. A “lisp” is an articulation problem that results in the ability to pronounce one or more consonants. There are 4 main types of lisps (inderdental, dentalized, lateral, and palatal). Some lisps are common and normal at various stages in development but should fade as children age. Lisps can be treated by an SLP with speech therapy.
Interdental lisp	Most common and well-known type of lisp, which is due to incorrect placement of the tongue within the mouth, with the tongue pushing forward between the front teeth. Most common is the inability to pronounce the sibilants /s/ or /z/, with production sounding like “th”. Called frontal distortion type I by Vallino and Tompson (1993) [48].
Dentalized lisp or dentalized production	The tongue tip pushes against the upper or lower anterior teeth (incisors), resulting in a muffled /s/ or /z/ sound. The tongue body is flattened, causing scattering of the air stream. Called frontal distortion type II by Vallino and Tompson (1993) [48].
Lateralized lisp	The air stream is diverted to one or both sides of the tongue, with air exiting the mouth out of the sides. This results in slushy or wet sounding speech, as speech is mixed with the sound of air mixing with saliva. Examples: Daffy Duck or Sylvester the Cat.
Palatal lisp	Least common type of lisp. Occurs when the center of tongue is in contact with the hard or soft palate, when attempting to produce the /s/ sound.
Whistling	High-frequency sound created by air passing between the tongue and alveolar ridge
Labiodentalization	Lower lip contacts the maxillary incisors

## Data Availability

No new data were created or analyzed in this study. Data sharing is not applicable to this article.

## References

[1] Fitch W The Evolution of Speech: A Comparative Review. Trends Cogn. Sci 2000, 4, 258–267. 10859570 10.1016/s1364-6613(00)01494-7

[2] Morgan TJH; Uomini NT; Rendell LE; Chouinard-Thuly L; Street SE; Lewis HM; Cross CP; Evans C; Kearney R; de la Torre I; Experimental Evidence for the Co-Evolution of Hominin Tool-Making Teaching and Language. Nat. Commun 2015, 6, 6029. 25585382 10.1038/ncomms7029 PMC4338549

[3] Blasi DE; Moran S; Moisik SR; Widmer P; Dediu D; Bickel B Human Sound Systems Are Shaped by Post-Neolithic Changes in Bite Configuration. Science 2019, 363, eaav3218. 30872490 10.1126/science.aav3218

[4] Ocampo-Parra A; Escobar-Toro B; Sierra-Alzate V; Rueda ZV; Lema MC Prevalence of Dyslalias in 8 to 16 Year-Old Students with Anterior Open Bite in the Municipality of Envigado, Colombia. BMC Oral Health 2015, 15, 77. 26160356 10.1186/s12903-015-0063-1 PMC4498501

[5] Hall BJC Attitudes of Fourth and Sixth Graders Toward Peers With Mild Articulation Disorders. Lang. Speech Hear. Serv. Sch 1991, 22, 334–340.

[6] Johnson CJ; Beitchman JH; Brownlie EB Twenty-Year Follow-up of Children with and without Speech-Language Impairments: Family, Educational, Occupational, and Quality of Life Outcomes. Am. J. Speech Lang. Pathol 2010, 19, 51–65. 19644128 10.1044/1058-0360(2009/08-0083)

[7] Mowrer DE; Wahl P; Doolan SJ Effect of Lisping on Audience Evaluation of Male Speakers. J. Speech Hear. Disord 1978, 43, 140–148. 661251 10.1044/jshd.4302.140

[8] Silverman EM Listeners’ Impressions of Speakers with Lateral Lisps. J. Speech Hear. Disord 1976, 41, 547–552. 994486 10.1044/jshd.4104.547

[9] Wadman R; Durkin K; Conti-Ramsden G Self-Esteem, Shyness, and Sociability in Adolescents with Specific Language Impairment (SLI). J. Speech Lang. Hear. Res 2008, 51, 938–952. 18658063 10.1044/1092-4388(2008/069)

[10] American Speech-Language-Hearing Association. Definitions of Communication Disorders and Variations; American Speech-Language-Hearing Association: Rockville, MD, USA, 1993.

[11] Proffit W; White R; Sarver D Contemporary Treatment of Dentofacial Deformity, 1st ed.; Mosby: St. Louis, MO, USA, 2003.

[12] Mendes de Paula Gomes A; Adas Saliba Garbin C; da Silva Ferraz FW; Adas Saliba T; Isper Garbin AJ Dentofacial Deformities and Implications on Quality of Life: A Presurgical Multifactorial Analysis in Patients Seeking Orthognathic Surgical Treatment. J. Oral Maxillofac. Surg 2019, 77, 409.e1. 10.1016/j.joms.2018.09.023 30352213

[13] Arya V; Kadagad P; Alvarez W; Chigurupati R; Mehra P Temporomandibular Disorders in Orthognathic Surgery Patients. J. Oral Maxillofac. Surg 2017, 75, e339–e340.

[14] Naini FB; Moss JP; Gill DS The Enigma of Facial Beauty: Esthetics, Proportions, Deformity, and Controversy. Am. J. Orthod. Dentofac. Orthop 2006, 130, 277–282. 10.1016/j.ajodo.2005.09.027 16979484

[15] Dujoncquoy J-P; Ferri J; Raoul G; Kleinheinz J Temporomandibular Joint Dysfunction and Orthognathic Surgery: A Retrospective Study. Head. Face Med 2010, 6, 27. 21083902 10.1186/1746-160X-6-27 PMC2998459

[16] Celakil D; Ozdemir F; Eraydin F; Celakil T Effect of Orthognathic Surgery on Masticatory Performance and Muscle Activity in Skeletal Class III Patients. CRANIO 2018, 36, 174–180. 28385103 10.1080/08869634.2017.1311395

[17] Uesugi T; Kobayashi T; Hasebe D; Tanaka R; Ike M; Saito C Effects of Orthognathic Surgery on Pharyngeal Airway and Respiratory Function during Sleep in Patients with Mandibular Prognathism. Int. J. Oral Maxillofac. Surg 2014, 43, 1082–1090. 25027545 10.1016/j.ijom.2014.06.010

[18] Migliorucci R; Abramides D; Rosa R; Bresaola M; Filho H; Berretin-Felix G Effect of Myofunctional Therapy on Orofacial Functions and Quality of Life in Individuals Undergoing Orthognathic Surgery. Int. J. Orofac. Myol. Myofunct. Ther 2017, 43, 60–76.

[19] Keyser MMB; Lathrop H; Jhingree S; Giduz N; Bocklage C; Couldwell S; Oliver S; Moss K; Frazier-Bowers S; Phillips C; Impacts of Skeletal Anterior Open Bite Malocclusion on Speech. FACE 2022, 3, 339–349. 35903399 10.1177/27325016221082229 PMC9328410

[20] Leavy KM; Cisneros GJ; LeBlanc EM Malocclusion and Its Relationship to Speech Sound Production: Redefining the Effect of Malocclusal Traits on Sound Production. Am. J. Orthod. Dentofac. Orthop 2016, 150, 116–123. 10.1016/j.ajodo.2015.12.015 27364213

[21] Knez Ambroži0č M; Hočevar Boltežar I; Ihan Hren N Changes of Some Functional Speech Disorders after Surgical Correction of Skeletal Anterior Open Bite. Int. J. Rehabil. Res 2015, 38, 246–252. 26164798 10.1097/MRR.0000000000000123

[22] Sahoo NK; Agarwal SS; Datana S; Bhandari SK Effect of Mandibular Advancement Surgery on Tongue Length and Height and Its Correlation with Upper Airway Dimensions. J. Maxillofac. Oral Surg 2020, 19, 624–629. 33071513 10.1007/s12663-020-01375-2 PMC7524986

[23] Tseng Y-C; Wu J-H; Chen C-M; Hsu K-J Correlation between Change of Tongue Area and Skeletal Stability after Correction of Mandibular Prognathism. Kaohsiung J. Med. Sci 2017, 33, 302–307. 28601235 10.1016/j.kjms.2017.03.008 PMC11916608

[24] Black LI; Vahratian A; Hoffman HJ Communication Disorders and Use of Intervention Services Among Children Aged 3–17 Years: United States, 2012; NCHS Data Brief; Centers for Disease Control and Prevention: Atlanta, GA, USA, 2015; pp. 1–8. 26079397

[25] Morris MA; Meier SK; Griffin JM; Branda ME; Phelan SM Prevalence and Etiologies of Adult Communication Disabilities in the United States: Results from the 2012 National Health Interview Survey. Disabil. Health J 2016, 9, 140–144. 26303018 10.1016/j.dhjo.2015.07.004

[26] Lathrop-Marshall H; Keyser MMB; Jhingree S; Giduz N; Bocklage C; Couldwell S; Edwards H; Glesener T; Moss K; Frazier-Bowers S; Orthognathic Speech Pathology: Impacts of Class III Malocclusion on Speech. Eur. J. Orthod 2022, 44, 340–351. 34562076 10.1093/ejo/cjab067 PMC9127721

[27] Yeni-Komshian GH; Kavanagh JF; Ferguson CA Speech Development in the First Year in Child Phonology; Academic Press: New York, NY, USA, 1980.

[28] Proffit W; Fields H; Larson B; Sarver D Contemporary Orthodontics, 6th ed.; Mosby: St Louis, MO, USA, 2018.

[29] Laitman JT; Reidenberg JS Specializations of the Human Upper Respiratory and Upper Digestive Systems as Seen through Comparative and Developmental Anatomy. Dysphagia 1993, 8, 318–325. 8269722 10.1007/BF01321770

[30] Cypreansen L Speech Development, Improvement, and Correction: Methods and Materials for the Classroom Teacher and the Speech Therapist; Ronald Press Co.: New York, NU, USA, 1959.

[31] Sander K When Are Speech Sounds Learned? J. Speech Hear. Disord 1972, 37, 55–63. 5053945 10.1044/jshd.3701.55

[32] Rogers H The Sounds of Language; Routledge: Abingdon, UK, 2014; ISBN 9781315838731.

[33] Rescorla L; Mirak J Normal Language Acquisition. Semin. Pediatr. Neurol 1997, 4, 70–76. 9195663 10.1016/s1071-9091(97)80022-8

[34] Cobourne M Orthodontic Management of the Developing Dentition; Cobourne MT, Ed.; Springer International Publishing: Cham, Switzerland, 2017; ISBN 978-3-319-54635-3.

[35] Gutierrez DS; Carugno P Thumb Sucking; StatPearls Publishing: Tampa, FL, USA, 2022. 32310572

[36] Arai T Physical Models of the Vocal Tract with a Flapping Tongue for Flap and Liquid Sounds. In Proceedings of the Interspeech 2013, ISCA, Lyon, France, 25–29 August 2013; pp. 2019–2023.

[37] Liu Y-P; Behrents RG; Buschang PH Mandibular Growth, Remodeling, and Maturation During Infancy and Early Childhood. Angle Orthod. 2010, 80, 97–105. 19852647 10.2319/020309-67.1 PMC8978730

[38] Currie K; Sawchuk D; Saltaji H; Oh H; Flores-Mir C; Lagravere M Posterior Cranial Base Natural Growth and Development: A Systematic Review. Angle Orthod. 2017, 87, 897–910. 28737426 10.2319/032717-218.1 PMC8317569

[39] Björk A; Skieller V Facial Development and Tooth Eruption. Mondo Ortod. 1977, 19, 29–63. 277776

[40] Jonasson G; Skoglund I; Rythén M The Rise and Fall of the Alveolar Process: Dependency of Teeth and Metabolic Aspects. Arch. Oral Biol 2018, 96, 195–200. 30292055 10.1016/j.archoralbio.2018.09.016

[41] Buschang PH; Roldan SI; Tadlock LP Guidelines for Assessing the Growth and Development of Orthodontic Patients. Semin. Orthod 2017, 23, 321–335.

[42] Lammert AC; Narayanan SS On Short-Time Estimation of Vocal Tract Length from Formant Frequencies. PLoS ONE 2015, 10, e0132193. 26177102 10.1371/journal.pone.0132193 PMC4503663

[43] Bennett BL Mosby’s Review Questions for the National Board Dental Hygiene Examination, 1st ed.; Mosby: St. Louis, MO, USA, 2014.

[44] Oliver S; Keyser MMB; Jhingree S; Bocklage C; Lathrop H; Giduz N; Moss K; Blakey G; White R; Turvey T; Impacts of Anterior-Posterior Jaw Disproportions on Speech of Dentofacial Disharmony Patients. Eur. J. Orthod 2023, 45, 1–10. 36308520 10.1093/ejo/cjac057 PMC9912703

[45] American Association of Orthodontists Why Orthodontics? Available online: https://www3.aaoinfo.org/blog/parent-s-guide-post/first-visit/ (accessed on 18 March 2023).

[46] MacLean JE; Fitzgerald DA; Waters KA Developmental Changes in Sleep and Breathing across Infancy and Childhood. Paediatr. Respir. Rev 2015, 16, 276–284. 26364005 10.1016/j.prrv.2015.08.002

[47] Kent RD Anatomical and Neuromuscular Maturation of the Speech Mechanism: Evidence from Acoustic Studies. J. Speech Hear. Res 1976, 19, 421–447. 979206 10.1044/jshr.1903.421

[48] Vallino LD; Tompson B Perceptual Characteristics of Consonant Errors Associated with Malocclusion. J. Oral Maxillofac. Surg 1993, 51, 850–856. 8336222 10.1016/s0278-2391(10)80101-6

[49] Johnson NC; Sandy JR Tooth Position and Speech–Is There a Relationship? Angle Orthod. 1999, 69, 306–310. 10456597 10.1043/0003-3219(1999)069<0306:TPASIT>2.3.CO;2

[50] Buyuknacar GB; Gulec A Correlation between the Cephalometric Measurements and Acoustic Properties of /s/ Sound in Turkish. J. Appl. Oral Sci 2020, 28, 1–8. 10.1590/1678-7757-2019-0399 PMC7185985 32348443

[51] Proffit W; White R; Reinhardt R Surgical Orthodontic Treatment, 1st ed.; Mosby: St. Louis, MO, USA, 1991.

[52] Ahn J; Kim G; Kim YH; Hong J Acoustic Analysis of Vowel Sounds before and after Orthognathic Surgery. J. Craniomaxillofac. Surg 2015, 43, 11–16. 25457743 10.1016/j.jcms.2014.10.002

[53] Bruce FA; Hanson ML Speech and Swallowing Changes Associated with Sagittal Osteotomy: A Report of Four Subjects. Int. J. Orofac. Myol 1987, 13, 1–6. 3479398

[54] Ghaemi H; Emrani E; Labafchi A; Famili K; Hashemzadeh H; Samieirad S The Effect of Bimaxillary Orthognathic Surgery on Nasalance, Articulation Errors, and Speech Intelligibility in Skeletal Class III Deformity Patients. World J. Plast. Surg 2021, 10, 8–14. 10.29252/wjps.10.1.8 PMC8016386 33833948

[55] Glass L; Knapp J; Bloomer HH Speech and Lingual Behavior before and after Mandibular Osteotomy. J. Oral Surg 1977, 35, 104–109. 264502

[56] Goodstein DB; Cooper D; Wallace L The Effect on Speech of Surgery for Correction of Mandibular Prognathism. Oral Surg. Oral Med. Oral Pathol 1974, 37, 846–849. 4524884 10.1016/0030-4220(74)90435-6

[57] Guay AH; Maxwell DL; Beecher R A Radiographic Study of Tongue Posture at Rest and during the Phonation of /s/ in Class III Malocclusion. Angle Orthod. 1978, 48, 10–22. 272127 10.1043/0003-3219(1978)048<0010:ARSOTP>2.0.CO;2

[58] Weimer K; Astrand P Effect on Speech of Mandibular Prognathism before and after Surgical Treatment. Swed. Dent. J 1977, 1, 173–176. 272067

[59] Garber SR; Speidel TM; Marse G The Effects on Speech of Surgical Premaxillary Osteotomy. Am. J. Orthod 1981, 79, 54–62. 6935972 10.1016/0002-9416(81)90101-9

[60] Niemi M; Laaksonen J-P; Peltomäki T; Kurimo J; Aaltonen O; Happonen R-P Acoustic Comparison of Vowel Sounds Produced before and after Orthognathic Surgery for Mandibular Advancement. J. Oral Maxillofac. Surg 2006, 64, 910–916. 16713805 10.1016/j.joms.2006.02.009

[61] Kravanja SL; Hocevar-Boltezar I; Music MM; Jarc A; Verdenik I; Ovsenik M Three-Dimensional Ultrasound Evaluation of Tongue Posture and Its Impact on Articulation Disorders in Preschool Children with Anterior Open Bite. Radiol. Oncol 2018, 52, 250–256. 30210041 10.2478/raon-2018-0032 PMC6137359

[62] Turvey TA; Journot V; Epker BN Correction of Anterior Open Bite Deformity: A Study of Tongue Function, Speech Changes, and Stability. J. Maxillofac. Surg 1976, 4, 93–101. 1065713 10.1016/s0301-0503(76)80014-8

[63] Bowers J; Tobey EA; Shaye R An Acoustic-Speech Study of Patients Who Received Orthognathic Surgery. Am. J. Orthod 1985, 88, 373–379. 3864371 10.1016/0002-9416(85)90064-8

[64] Dalston RM; Vig PS Effects of Orthognathic Surgery on Speech: A Prospective Study. Am. J. Orthod 1984, 86, 291–298. 6592977 10.1016/0002-9416(84)90139-8

[65] Geffen D The Effects of Mandibular Osteotomy on Articulation and Resonance. S. Afr. J. Commun. Disord 1978, 25, 54–62. 749219 10.4102/sajcd.v25i1.372

[66] Laine T Malocclusion Traits and Articulatory Components of Speech. Eur. J. Orthod 1992, 14, 302–309. 1516663 10.1093/ejo/14.4.302

[67] Lichnowska A; Kozakiewicz M The Logopedic Evaluation of Adult Patients after Orthognathic Surgery. Appl. Sci 2021, 11, 5732.

[68] Ruscello DM; Tekieli ME; Jakomis T; Cook L; Van Sickels JE The Effects of Orthognathic Surgery on Speech Production. Am. J. Orthod 1986, 89, 237–241. 3456716 10.1016/0002-9416(86)90038-2

[69] Vallino LD Speech, Velopharyngeal Function, and Hearing before and after Orthognathic Surgery. J. Oral Maxillofac. Surg 1990, 48, 1274–1281. 2231145 10.1016/0278-2391(90)90481-g

[70] Wakumoto M; Isaacson KG; Friel S; Suzuki N; Gibbon F; Nixon F; Hardcastle WJ; Michi K Preliminary Study of Articulatory Reorganisation of Fricative Consonants Following Osteotomy. Folia Phoniatr. Logop 1996, 48, 275–289. 8958664 10.1159/000266422

[71] Ward EC; McAuliffe M; Holmes SK; Lynham A; Monsour F Impact of Malocclusion and Orthognathic Reconstruction Surgery on Resonance and Articulatory Function: An Examination of Variability in Five Cases. Br. J. Oral Maxillofac. Surg 2002, 40, 410–417. 12379188

[72] Witzel MA; Ross RB; Munro IR Articulation before and after Facial Osteotomy. J. Maxillofac. Surg 1980, 8, 195–202. 6932461 10.1016/s0301-0503(80)80100-7

[73] Amr-Rey O; Sánchez-Delgado P; Salvador-Palmer R; Cibrián R; Paredes-Gallardo V Association between Malocclusion and Articulation of Phonemes in Early Childhood. Angle Orthod. 2022, 92, 505–511. 35275982 10.2319/043021-342.1 PMC9235385

[74] Klechak TL; Bradley DP; Warren DW Anterior Open Bite and Oral Port Constriction. Angle Orthod. 1976, 46, 232–242. 1066975 10.1043/0003-3219(1976)046<0232:AOBAOP>2.0.CO;2

[75] O’Gara M; Wilson K The Effects of Maxillofacial Surgery on Speech and Velopharyngeal Function. Clin. Plast. Surg 2007, 34, 395–402. 17692699 10.1016/j.cps.2007.04.001

[76] Habel A; Sell D; Mars M Cleft Palate Speech Management. In The Dynamics of Speech and Orthodontic Management in Cleft Lip and Palate; Mosby: St. Louis, MO, USA, 1995; pp. 352–363.

[77] Vig KW; Fields HW Facial Growth and Management of Orthodontic Problems. Pediatr. Clin. N. Am 2000, 47, 1085–1123. 10.1016/s0031-3955(05)70259-5 11059351

[78] Farronato G; Giannini L; Riva R; Galbiati G; Maspero C Correlations between Malocclusions and Dyslalias. Eur. J. Paediatr. Dent 2012, 13, 13–18. 22455522

